# Grit and Meaning in Life of Chinese Nurses: The Chain Mediating Effect of Social Support and Hope

**DOI:** 10.3389/fpsyg.2021.769707

**Published:** 2021-11-11

**Authors:** Lei Yang, Dongmei Wu

**Affiliations:** ^1^School of Nursing, Chengdu Medical College, Chengdu, China; ^2^Department of Nursing, The Clinical Hospital of Chengdu Brain Science Institute, MOE Key Laboratory for Neuroinformation, University of Electronic Science and Technology of China, Chengdu, China

**Keywords:** grit, social support, hope, meaning in life, China, nurses

## Abstract

Grit is defined as perseverance and passion for long-term goals, and it may affect the stability of the nursing workforce and the physical and mental health of nurses continuously. Meaning in life has received considerable attention from scholars, which is an important component in positive psychology. This study aimed to delve into the relationship between grit and the meaning in life of Chinese nurses. Additionally, we also sought to prove the chain mediating effect of social support and hope on this relationship. An online questionnaire survey was used to collect data from 704 Chinese nurses using the self-made demographic questionnaire with Short Grit Scale (Grit-S), the Perceived Social Support Scale (PSSS), Adult Dispositional Hope Scale (ADHS), and Meaning in Life Questionnaire (MLQ). Moreover, Process version 3.3 plug-in SPSS 25 was used to test the mediation effect between variables. The results showed a strong positive relationship between grit and meaning in life and verified the mediating effect of social support and hope on grit and meaning in life. The results also confirmed the chain mediating model between grit, social support, hope, and meaning in life.

## Introduction

Since the 1950s, positive psychology has become increasingly prominent among scholars. Researchers pointed out that positive psychology should pay attention to positive mental status (i.e., positive emotions, positive environments, and positive attitudes) and other related concepts (Seligman et al., 2005; Moskowitz et al., 2019). Meaning in life, related to the ability of an individual to survive in the face of adversity, is a basic element of human existence (Frankl, 1966). Steger et al. (2008) noted that the key to meaning in life is to be aware of the current goal and ultimately achieve it. Nursing plays a paramount role in saving lives and promoting the health status of patients, as well as being the frontline workers in global health emergencies like the outbreak of the covid-19 pandemic. High role expectations and varieties of stressors caused a negative impact on the development of the careers of nurses (Tyer-Viola, 2019). Based on numerous demands for nurses, they are required to have a greater sense of responsibility and mission. Therefore, nurses must own a clear life goal, and strive to enhance the sense of meaning in life. The study of Czekierda et al. (2017) identified meaning in life as a potential role of physical health, with a mild to moderate positive impact. More studies had confirmed that meaning in life is positively correlated with the quality of life and self-esteem (Barnett et al., 2019; Bernard et al., 2020). Furthermore, meaning in life is an important predictor of subjective well-being (Krok and Gerymski, 2019) and is related to positive emotions, life satisfaction, and career decision self-efficacy (Sari, 2019). Meanwhile, researchers showed an insufficient sense of meaning in life may experience stressor-related distresses suicidal tendencies, and negative thinking (Barnett et al., 2019; Lew and Chistopolskaya, 2020; Ostafin and Proulx, 2020).

### The Impact of Grit on Meaning in Life

When encountering challenges or disasters, nurses need to delve into high-quality patient care, retain resilience, and process professionally in the face of adversity continually (Brennan, 2017). Generally, a personality trait named grit drives nurses to complete the mission on difficult occasions (Meyer et al., 2020). Gritty people always adopt various strategies to achieve long-term goals and alleviate negative impacts in life (Blalock et al., 2015). Duckworth and Quinn (2009) categorized grit into consistent interest and persistent effort. The existing studies had confirmed that grit is an important non-cognitive trait of success or failure among nursing students (Terry and Peck, 2020), nurses (Tyer-Viola, 2019), or nurse leaders (Seguin, 2019). Grit was a significant predictor of nursing students in clinical and academic achievement, regardless of demographic factors (Terry and Peck, 2020). Researchers demonstrated that grit was associated with increased job involvement, greater career longevity, lowered turnover intention, and improved well-being for nurses and nurse leaders (Jeong and Seo, 2019; Seguin, 2019). Therefore, the study tried to hypothesize the following:

*Hypothesis 1: Grit will significantly predict meaning in life (grit*→ *meaning in life).*

### The Mediating Role of Social Support Between Grit and Meaning in Life

Perceived social support implies that individuals experience a subjective feeling of being understood, respected, and supported by others (Liu and Aungsuroch, 2019). Social support was categorized as external (from friends and family) and internal (from colleagues and leaders) support (Hamama et al., 2019). Social support was regarded as motivation that improved job satisfaction and alleviated the work stress of nurses successfully (Orgambídez and Almeida, 2020). In the study of Nowicki and Ślusarska (2020), they indicated that peer support is an important risk factor for traumatic stress among nurses, especially facing major public health events. The work of Krause and Rainville (2020) suggested that social support mediated the relationship between age and meaning in life. In another study, Lee et al. (2017) discovered a significant relationship between the search for meaning and perceived social support. When receiving more social support, nurses can cope with the negative effects of stressful environments, stimulate enthusiasm for work, and improve their sense of meaning in life. Therefore, the study tried to hypothesize the following:

*Hypothesis 2: Social support will act as a mediator between grit and meaning in life (grit*→ *social support*→ *meaning in life).*

### The Mediating Role of Hope Between Grit and Meaning in Life

Hope is a positive expectation of results (Scioli et al., 2011) and plays a vital role in clinical practice (Zhang et al., 2020). The broaden-and-built theory of positive emotions showed that hope can reduce the influence of negative emotions by constructing psychological resources (Fredrickson, 2001). Numerous studies have shown that hope reduces psychological distress, maintains adaptation to disease, improves well-being, and driving directions for existence (Mattioli et al., 2008; Rustøen et al., 2011). In addition, a high level of personal hope can divert their attention from negative events (Kaleta and Justyna, 2020), reduce the risk of depression and suicide, and improve meaning in life (Sun et al., 2021). Therefore, the study tried to hypothesize the following:

*Hypothesis 3: Hope will act as a mediator between grit and meaning in life (grit*→ *hope*→ *meaning in life).*

### The Chain Mediating Role of Social Support and Hope on Grit and Meaning in Life

Many studies have suggested grit, social support, hope, and meaning in life were positively correlated with quality of life, job satisfaction, and well-being (Seguin, 2019; Bernard et al., 2020; Orgambídez and Almeida, 2020; Shiri et al., 2020). Nurses with grit can be combined with positive environmental factors represented by social support and positive attitudes represented by hope to tolerate stress, and improve quality of life, job satisfaction, subjective well-being, and finally enhance the sense of meaning in life. Therefore, the study tried to explore the following:

*Hypothesis 4: Social support and hope will jointly act a chain mediator role in the relationship between grit and meaning in life (grit*→ *social support*→ *hope*→ *meaning in life).*

Although previous studies have separately explored the relationship between grit, social support, hope, and meaning in life, few studies have shown how grit affects the meaning in life through the mediating role of social support and hope. Generally, only a few works of literature have focused on personality traits and meaning in the life of hospice nurses (Barnett et al., 2019), oncology nurses (Candela and Piredda, 2020), and pediatric nurses (Taubman-Ben-Ari and Weintroub, 2008). Therefore, this study intended to delve into the impact of the grit of Chinese nurses on the meaning in life from the perspective of positive psychology and analyze the mediating role of social support.

## Materials and Methods

### Participants

Since data collection was conducted at one specific time, the nature of this study was cross-sectional. The study group consisted of 756 nurses who were recruited from different hospitals in Chengdu City, China, who met criteria through an online questionnaire survey. Among the 756 nurses, 704 of whom were valid (52 nurses failed to complete all answers). The response rate was 93.12%. All participants had signed informed consent and voluntarily participated in this study. The inclusion criteria were: (a) obtained professional qualification certificate in the People’s Republic of China; (b) have at least 1-year working experience in clinical nursing or clinical nursing management; (c) no previous or current diagnosis of mental illness or drug or alcohol dependence; (d) had a basic phone or computer skills; (e) informed to participate in the study. Nurses who failed to complete the survey were excluded. The characteristics of the participants are shown in Table 1.

**TABLE 1:** Baseline characteristics and difference in the grit score of nurses.

**Variables**	**All sample (*n* = 704)**	**Grit scores (M ± SD)**	***t/F***	***P***
Gender			*t* = 1.618	0.106
Male	66 (9.4%)	27.85 ± 4.27		
Female	638 (90.6%)	26.99 ± 4.10		
Age(years)			*t* = −1.057	0.291
<30	315 (44.74%)	26.89 ± 4.18		
≥30	389 (55.26%)	27.22 ± 4.07		
Educational background			*t* = 0.876	0.381
1 College degree	302 (42.9%)	27.23 ± 3.97		
Bachelor or graduate degree	402(57.1%)	26.95 ± 4.23		
Length of nursing work			*t* = 0.028	0.978
<10	403 (57.24%)	27.07 ± 4.07		
≥10	301 (42.76%)	27.06 ± 4.19		

*SD = standard deviation.*

### Measures

All tests were conducted in Mandarin Chinese.

#### Demographic Variables

A self-made demographic questionnaire was utilized in this study to collect the characteristics of participants, including gender (male, female), age, educational background (college degree, bachelor’s, or graduate degree), and length of nursing work.

#### Grit

The self-reported Short Grit Scale (Grit-S) was developed and validated by Duckworth and Quinn (2009) including two subscales, namely, consistency of interest and perseverance of effort. A total of eight items in the scale were included and used the 5-point Likert scale (from 1 = “not like me at all” to 5 = “very much like me”). The Grit-S of the Chinese version has good reliability which had been verified by Li et al. (2018). The Cronbach’s α of the self-reported scale was 0.73–0.83 in a previous study (Duckworth and Quinn, 2009). In addition, the Cronbach’s α of subscales were from 0.58 to 0.71 in China (Wang, 2016).

#### Social Support

The 12-item Perceived Social Support Scale (PSSS) was developed and validated by Zimet et al. (1990), including three subscales (family support, friends support, and other support). Participants rated on a 7-point Likert response format (from 1 = “very strongly disagree” to 7 = “very strongly agree”). The total scores ranged from 12 to 84, with higher scores suggesting greater perceived social support. The Cronbach’s α for the PSSS was 0.914 in the current study (Liu et al., 2016).

#### Hope

The Adult Dispositional Hope Scale (ADHS) was originally designed by Snyder et al. (1991) and the Chinese version was applied and verified by Ren (2006). The ADHS may be applied to individuals older than 15 years to assess the dispositional level of hope. The scale consisted of 12 items, wherein 4 items (items 3, 5, 7, 11) served as fillers and were not interpreted. The other 8 items were divided into 2 dimensions, measuring pathways thinking (items 1, 4, 6, 8) and agency (items 2, 9, 10, 12). ADHS adopted a 4-point scoring method (from 1 = “definitely false” to 4 = “definitely true”). The Cronbach’s α of the scales was from 0.74 to 0.84 (Snyder et al., 1991).

#### Meaning in Life

The 10-item Meaning in Life Questionnaire (MLQ) was originally compiled by Steger et al. (2008), including two subscales, namely, the presence of meaning and the search for meaning. The Chinese version of MLQ was revised and verified by Wang et al. (2016). Participants rated on a seven-point Likert response format (from 1 = “completely inconsistent” to 7 = “completely consistent”). The Cronbach’s α of the two subscales was 0.88 and 0.93, respectively (Steger et al., 2009).

#### Procedure

The study was ethically approved by the Ethics Committee of Chengdu 4th Hospital and the registration number of the Chinese Clinical Trial Registry is ChiCTR1900020715. Nurses were asked to complete an anonymous online survey. Each hospital appointed an investigating nurse who had been trained by researchers previously. Participants had signed informed consent before the investigation and voluntarily participated in this study. Investigating nurse is requested to distribute the questionnaire to other nurses. Subsequently, nurses were invited to click on a web link^1^ to access the questionnaire *via* mobile phones. The investigating nurses explained the unclear and ambiguous items suggested by the participants during the field investigation according to the unified guidelines. Note that all questionnaires were self-rated, and participants filled separately.

### Statistical Analysis

The following data analyses were used to verify the relationship between grit and meaning in life and verify the mediating effect of social support, and hope on grit and meaning in life. Note that the statistical description of the count data was represented by the composition ratio [n (%)], while the measurement data conforming to the normal distribution was represented by (*M* ± *SD*). Comparison between groups was represented by the *t*-test. Pearson correlation analysis was adopted between both factors of grit, social support, hope, and meaning in life. Process V3.3 in SPSS (IBM. V25.0) was used to analyze the mediating effect of social support and hope on grit and meaning in the life of clinical nurses. The bootstrap method was used to estimate the 95% confidence interval with 5,000 repeated sampling, and Two-sided inspection level α = 0.05.

## Results

A total of 756 nurses were recruited in this study, with 704 nurses satisfying the necessary criteria. A majority of nurses were female (90.6%) and their average age was 31.79 (*SD* = 7.38) years. Almost half of the nurses (57.1%) possessed a bachelor’s or graduate degree. Their average length of nursing work was 10.72 (*SD* = 8.12) years (Table 1). However, there were no significant differences in the grit scores of nurses among different subgroups of gender, age, educational background, and length of nursing work (*P* > 0.05) (Table 1).

### Correlation Analysis of Major Study Variables

Pearson correlation analysis showed that grit was positively related to social support (*r* = 0.407, *P* < 0.01), hope (*r* = 0.506, *P* < 0.01), and meaning in life (*r* = 0.455, *P* < 0.01). Similarly, social support was positively correlated with hope (*r* = 0.484, *P* < 0.01) and meaning in life (*r* = 0.546, *P* < 0.01). In addition, hope was positively related to meaning in life (*r* = 0.589, *P* < 0.01) (Table 2).

**TABLE 2:** Correlation analysis of study variables.

**Variables**	***Mean***	***SD***	**1**	**2**	**3**	**4**
1. Grit	27.03	4.14	–			
2. Social support	42.82	6.56	0.407**	–		
3. Hope	21.94	2.96	0.506**	0.484**	–	
4. Meaning in life	21.07	4.24	0.455**	0.546**	0.589**	–

*SD = standard deviation.*

**P < 0.01.

### Multiple Mediating Analyses Between Variables of Clinical Nurses

The results showed the total effect (β = 0.456, *t* = 13.5, *P* < 0.001) and the direct effect (β = 0.142, *t* = 4.251, *P* < 0.001) of grit on the meaning in life were both significant, after controlling the variables such as gender, educational background, and length of nursing work. Grit significantly predicts social support (β = 0.408, *t* = 11.827, *P* < 0.001) and social support predicts meaning in life (β = 0.311, *t* = 9.402, *P* < 0.001), indicating that social support played a mediating role between grit and meaning in life. Similarly, grit significantly predicts hope (β = 0.367, *t* = 11.107, *P* < 0.001) and hope predicts meaning in life (β = 0.37, *t* = 10.459, *P* < 0.001) indicating that hope played a mediating role between grit and meaning in life. Meanwhile, social support can also predict hope (β = 0.338, *t* = 10.223, *P* < 0.001). Therefore, social support and hope had a chain mediating effect between grit and the meaning in the life of Chinese nurses (Table 3).

**TABLE 3:** Regression model of the effect of grit on meaning in life among Chinese nurses.

**Variables**	**β**	***t***	***P***	***LLCI***	***ULCI***	***R* ^2^**	***F***
**Step 1 Outcome variable: Social support**							
Predictor grit	0.408	11.827	<0.001	0.341	0.476	0.172	29.647
**Step 2 Outcome variable: Hope**							
Predictor grit	0.367	11.107	<0.001	0.302	0.432	0.369	67.910
Mediator social support	0.338	10.223	<0.001	0.273	0.403		
**Step 3 Outcome variable: Meaning in life**							
Predictor grit	0.142	4.250	<0.001	0.077	0.208	0.452	81.884
Mediator 1 Social support	0.311	9.402	<0.001	0.246	0.376		
Mediator 2 Hope	0.370	10.459	<0.001	0.300	0.439		
**Step 4 Outcome variable: Meaning in life**							
Independent variable grit	0.456	13.500	<0.001	0.390	0.522	0.208	36.679

**FIGURE 1:**
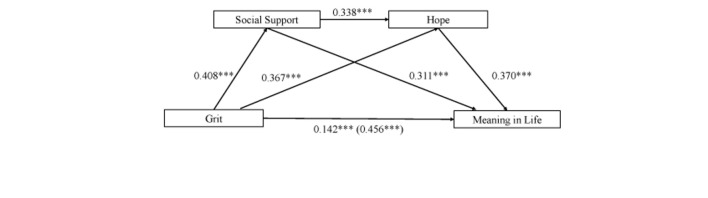
Chain mediating model (^∗∗∗^*P* < 0.01).

Results of the mediating effect analysis in Table 4 showed that Bootstrap’s 95% CI of total indirect effect did not contain 0 [Bootstrap 95% CI:0.258,0.374], accounting for 68.86% of the total effect. Importantly, three indirect effect pathways influenced the relation of grit and meaning in life. Firstly, mediating effect value of Path1 (Grit → Social Support → Meaning in Life) was 0.127 [Bootstrap 95% CI:0.09,0.166], accounting for 27.85% of the total effect. Secondly, mediating effect value of Path2 (Grit → Hope → Meaning in Life) was 0.136 [Bootstrap 95% CI:0.100,0.176], accounting for 29.82% of the total effect. Thirdly, the mediating effect value of Path3 (Grit → Social Support → Hope → Meaning in Life) was 0.051 [Bootstrap 95% CI:0.034,0.072], accounting for 11.18% of the total indirect effect. Note that the Chain mediating model is shown in Figure 1.

**TABLE 4:** Multiple mediated analysis between variables of nurses.

	**Effect**	**Boot SE**	**Bootstrap 95% CI**		**Effect ratio**
			**Low**	**High**	
Total effect	0.456	0.034	0.390	0.522	100%
Direct effect	0.142	0.034	0.077	0.208	31.14%
Total indirect effect	0.314	0.030	0.258	0.374	68.86%
Path1: Grit→ Social Support→ Meaning in Life	0.127	0.019	0.090	0.166	27.85%
Path2: Grit→ Hope→ Meaning in Life	0.136	0.020	0.100	0.176	29.82%
Path3: Grit→ Social Support→ Hope→ Meaning in Life	0.051	0.010	0.034	0.072	11.18%
Comparsion1 (Path1 and Path2)	–0.009	0.031	–0.067	0.053	
Comparsion2 (Path1 and Path3)	0.076	0.020	0.040	0.116	
Comparsion3 (Path2 and Path3)	0.085	0.020	0.047	0.126	

Pairwise comparison of the different indirect effects paths was adopted to verify whether these paths were significant different. The results showed that Comparison 2 [Bootstrap 95% CI:0.04,0.116] and 3 [Bootstrap 95% CI:0.047,0.116] were significant, except Comparison 1 [Bootstrap 95% CI: –0.067,0.053].

## Discussion

A rigorous perusal of existing literature revealed that limited studies have investigated the relationship and specific pathways between grit and meaning in life among Chinese nurses. The present study adopted a chain mediating model to explore the influence of grit, social support, and hope on meaning in life in Chinese nurses. The results supported the hypotheses and verified the mediating role of social support and hope in the relationship between grit and meaning in life.

Interestingly, a key finding of this study was grit had a significant positive effect on meaning in life, after controlling for gender, educational background, and length of nursing work. These results strengthen the relationship between grit and meaning in life (Oriol et al., 2020). We extended this conclusion to Chinese nurses. Nurses were increasing due to the development of health services in China (Zhao and Zhang, 2015). While nurses were confronted with more and more work and psychological pressure because of the huge influx of illness patients, workplace violence (Lu et al., 2019), and insufficient resources for health services, such as shortage of beds and the imbalance between medical staff and patients (Zeng et al., 2013). Individuals with higher grit do not seek immediate satisfaction and success but strive to achieve their goals after years of unswerving efforts (Duckworth et al., 2007). In the study of Sharkey et al. (2017), they indicated that grit positively correlated to health-related life quality among adolescents and young adults. In addition, there was a positive relationship between grit and life satisfaction, with self-esteem as the mediator variable in employees (Li et al., 2018). The work of Vainio and Daukantaitë (2016) implied that grit was positively related to well-being and mediated by a sense of coherence and authenticity. Thus, grit could play a positive role in promoting quality of life and meaning in life.

Furthermore, social support, hope, and meaning in life were both positively correlated, had been proven in many studies (Mahon and Yarcheski, 2017; Cao and Zhou, 2021; Torregrosa-Ruiz et al., 2021). The work of Bruhn and Philips (1987) noted individuals generated hope by seeking help from trustworthy people, which meant hope was given and received in a supportive relationship. Similarly, Russinova (1999) stated hope occurred with the support of family, parents, friends, and peers. The study of Lin et al. (2020) suggested that social support and meaning in life were associated with the life satisfaction of people. Researchers indicated people with social support and the presence of meaning in life were fewer negative effects and were less susceptible to mental illness (Lin et al., 2020; Cao and Zhou, 2021). Karataş et al. (2021) pointed that meaning in life and hope were significant predictors of life satisfaction. In addition, Snyder (2002) stated that hope, as an important psychological fore, turned stress into motivation with a positive impact on life satisfaction.

Another important finding is the chain mediating effect of social support and hope in the relationship between grit and meaning in the life of Chinese nurses. This is the main theoretical contribution of this research. The results showed grit indirectly affects meaning in life through three pathways: social support, hope, and the chain mediating effect of social support and hope. Previous studies demonstrated the partial mediating effect of social support in the relationship between resilience and the quality of life (Zhang et al., 2017). The current study demonstrated social support played a partial mediating role in the correlation between grit and the meaning in life (Path 1), accounting for 27.85% of the total indirect effect, which means that grit and social support are important predictors in the meaning of life. Social support is one of the most common positive external sources for coping with negative psychological events and has been widely cited as a protective factor of psychosocial adaptation (Oexle and Sheehan, 2020). The study also confirmed the mediating effect of hope underlying grit and meaning in life relationships (Path 2), accounting for 29.82% of the total indirect effect. It can be found that the mediating role of hope is greater than social support. Hope is a vital psychological resource, which is of great significance for building values and achieving success. Nurses with brave and gritty can increase motivation to attach goals, raise the level of hope, and eventually generate a sense of meaning. Finally, there existed another insignificant path (grit → social support → hope → meaning in life) (Path 3), accounting for 11.18% of the total indirect effect, which showed grit influenced the meaning in life through social support and hope. Social support and hope jointly promote meaning in life. Under tremendous work and psychological pressure, improvement of the sense of meaning in life contributes to reduce negative emotions and improve the quality of life and well-being of nurses.

The study enriches the content of positive psychology and deeply explores the mechanism of grit on meaning in life from the perspective of Chinese nurses. It turns out that social support and hope have an important role in the relationship, which provides a useful reference for the construction of a structural model of grit in the future.

## Conclusion

Grit is an essential trait for the challenging profession of nursing. Some insights were provided into the relationship between grit and meaning in the life of Chinese nurses. This study demonstrated the relationship between grit and the meaning in the life of Chinese nurses and the mediate effect of social support and hope on grit and meaning in life. Specifically, the results gave evidence to infer that grit has a positive correlation with social support, hope, and meaning in life. Moreover, grit might directly or indirectly affect meaning in life through social support and hope. Based on the mediate effects, Path 1 and Path 2 played an important role in the total indirect effect. In conclusion, grit positively affected Chinese nurses to increase their levels of social support, hope, and meaning in life.

## Limitations

There were several limitations in the current study. Firstly, the results were based on an online self-reported survey, which was highly prone to inaccurate and biased responses from participants. Secondly, this study was cross-sectional in design and impossible to clarify the causal relationship between the variables. Longitudinal research should be designed to supplement and verify the validity and reliability of our findings. Thirdly, the mediation variables in this research were social support and hope, while other variables might mediate grit and meaning in life. Finally, interactions between social support and hope were not analyzed and reported in this research.

## Data Availability Statement

The original contributions presented in the study are included in the article/supplementary material, further inquiries can be directed to the corresponding author.

## Ethics Statement

The studies involving human participants were reviewed and approved by Committee of Chengdu 4th Hospital. The patients/participants provided their written informed consent to participate in this study.

## Author Contributions

LY was involved in all aspects of the study and preparation of the manuscript. DW was involved with the design of the study and preparation of the manuscript.

## Conflict of Interest

The authors declare that the research was conducted in the absence of any commercial or financial relationships that could be construed as a potential conflict of interest.

## Publisher’s Note

All claims expressed in this article are solely those of the authors and do not necessarily represent those of their affiliated organizations, or those of the publisher, the editors and the reviewers. Any product that may be evaluated in this article, or claim that may be made by its manufacturer, is not guaranteed or endorsed by the publisher.

## Footnotes

1
http://www.wjx.cn/

## References

[B1] Barnett M. D. Moore J. M. Garza C. J. (2019). Meaning in life and self-esteem help hospice nurses withstand prolonged exposure to death. *J. Nurs. Manag.* 27 775–780. 10.1111/jonm.12737 30481407

[B2] Bernard M. Berchtold A. Strasser F. Gamondi C. (2020). Meaning in life and quality of life: palliative care patients versus the general population. *BMJ Support Palliat Care* 6:bmjspcare-2020-002211. 10.1136/bmjspcare-2020-002211 32631960 PMC11671900

[B3] Blalock D. V. Young K. C. Kleiman E. M. (2015). Stability amidst turmoil: grit buffers the effects of negative life events on suicidal ideation. *Psychiatry Res.* 228 781–784. 10.1016/j.psychres.2015.04.041 26070767

[B4] Brennan E. J. (2017). Towards resilience and wellbeing in nurses. *Br. J. Nurs.* 26 43–47. 10.12968/bjon.2017.26.1.43 28079412

[B5] Bruhn J. G. Philips B. U. (1987). A developmental basis for social support. *J. Behav. Med.* 10 213–229. 10.1007/BF00846536 3612779

[B6] Candela M. L. Piredda M. (2020). Finding meaning in life: an exploration on the experiences with dependence on care of patients with advanced cancer and nurses caring for them. *Support Care Cancer* 28 4493–4499. 10.1007/s00520-020-05300-8 31942641

[B7] Cao Q. Zhou Y. (2021). Association between social support and life satisfaction among people with substance use disorder: the mediating role of resilience. *J. Ethn. Subst. Abuse* 20 415–427. 10.1080/15332640.2019.1657545 31544654

[B8] Czekierda K. Banik A. Park C. L. Luszczynska A. (2017). Meaning in life and physical health: systematic review and meta-analysis. *Health Psychol. Rev.* 11 387–418. 10.1080/17437199.2017.1327325 28488471

[B9] Duckworth A. L. Peterson C. Matthews M. D. Kelly D. R. (2007). Grit: perseverance and passion for long-term goals. *J. Pers. Soc. Psychol.* 92 1087–1101. 10.1037/0022-3514.92.6.1087 17547490

[B10] Duckworth A. L. Quinn P. D. (2009). Development and validation of the short grit scale (grit-s). *J. Pers. Assess.* 91 166–174. 10.1080/00223890802634290 19205937

[B11] Frankl V. E. (1966). Logotherapy and existential analysis–a review. *Am. J. Psychother.* 20 252–260. 10.1176/appi.psychotherapy.1966.20.2.252 5327827

[B12] Fredrickson B. L. (2001). The role of positive emotions in positive psychology. The broaden-and-build theory of positive emotions. *Am. Psychol.* 56 218–226. 10.1037//0003-066x.56.3.218 11315248 PMC3122271

[B13] Hamama L. Hamama-Raz Y. Stokar Y. N. Pat-Horenczyk R. Brom D. Bron-Harlev E. (2019). Burnout and perceived social support: the mediating role of secondary traumatization in nurses vs. physicians. *J. Adv. Nurs.* 75 2742–2752. 10.1111/jan.14122 31231845

[B14] Jeong J. Y. Seo Y. S. (2019). The Influence of grit on turnover intention of university hospital nurses: the mediating effect of job involvement. *J. Korean Acad. Nurs.* 49 181–190. 10.4040/jkan.2019.49.2.181 31064971

[B15] Kaleta K. Justyna M. (2020). The relationship between basic hope and depression: forgiveness as a mediator. *Psychiatr. Q.* 91 877–886. 10.1007/s11126-020-09759-w 32361795 PMC7395009

[B16] Karataş Z. Uzun K. Tagay Ö (2021). Relationships between the life satisfaction, meaning in life, hope and COVID-19 fear for Turkish adults during the COVID-19 outbreak. *Front. Psychol.* 12:633384. 10.3389/fpsyg.2021.633384 33776856 PMC7990893

[B17] Krause N. Rainville G. (2020). Age differences in meaning in life: exploring the mediating role of social support. *Arch. Gerontol. Geriatr.* 88:104008. 10.1016/j.archger.2020.104008 32058124

[B18] Krok D. Gerymski R. (2019). Self-efficacy as a mediator of the relationship between meaning in life and subjective well-being in cardiac patients. *Curr. Issues Pers. Psychol.* 7 242–251. 10.5114/cipp.2019.89168

[B19] Lee S. H. Nam H. S. Kim H. B. Kim E. J. Won S. D. Chae J. H. (2017). Social support as a mediator of posttraumatic embitterment and perceptions of meaning in life among Danwon survivors of the Sewol Ferry disaster. *Yonsei Med. J.* 58 1211–1215. 10.3349/ymj.2017.58.6.1211 29047246 PMC5653487

[B20] Lew B. Chistopolskaya K. (2020). Meaning in life as a protective factor against suicidal tendencies in Chinese University students. *BMC Psychiatry* 20:73. 10.1186/s12888-020-02485-4 32070298 PMC7027298

[B21] Li J. Fang M. Wang W. Sun G. Cheng Z. (2018). The Influence of grit on life satisfaction: self-esteem as a mediator. *Psychol. Belg.* 58 51–66. 10.5334/pb.400 30479807 PMC6194520

[B22] Lin Y. Xiao H. Lan X. Wen S. Bao S. (2020). Living arrangements and life satisfaction: mediation by social support and meaning in life. *BMC Geriatr.* 20:136. 10.1186/s12877-020-01541-8 32293305 PMC7158054

[B23] Liu L. Gou Z. Zuo J. (2016). Social support mediates loneliness and depression in elderly people. *J. Health Psychol.* 21 750–758. 10.1177/1359105314536941 24925547

[B24] Liu Y. Aungsuroch Y. (2019). Work stress, perceived social support, self-efficacy and burnout among Chinese registered nurses. *J. Nurs. Manag.* 27 1445–1453. 10.1111/jonm.12828 31306524

[B25] Lu L. Lok K. I. Zhang L. Hu A. Ungvari G. S. Bressington D. T. (2019). Prevalence of verbal and physical workplace violence against nurses in psychiatric hospitals in China. *Arch. Psychiatr. Nurs.* 33 68–72. 10.1016/j.apnu.2019.07.002 31711597

[B26] Mahon N. E. Yarcheski A. (2017). Parent and friend social support and adolescent hope. *Clin. Nurs. Res.* 26 224–240. 10.1177/1054773815619881 26655561

[B27] Mattioli J. L. Repinski R. Chappy S. L. (2008). The meaning of hope and social support in patients receiving chemotherapy. *Oncol. Nurs. Forum* 35 822–829. 10.1188/08.onf.822-829 18765329

[B28] Meyer G. Shatto B. Kuljeerung O. Nuccio L. Bergen A. Wilson C. R. (2020). Exploring the relationship between resilience and grit among nursing students: a correlational research study. *Nurse Educ. Today* 84:104246. 10.1016/j.nedt.2019.104246 31706204

[B29] Moskowitz J. T. Addington E. L. Cheung E. O. (2019). Positive psychology: a personal history. *Ann. Rev. Clin. Psychol.* 15 1–23. 10.1016/j.genhosppsych.2019.11.001 30525996

[B30] Nowicki G. J. Ślusarska B. (2020). The severity of traumatic stress associated with COVID-19 pandemic, perception of support, sense of security, and sense of meaning in life among nurses: research protocol and preliminary results from Poland. *Int. J. Environ. Res. Public Health* 17:6491. 10.3390/ijerph17186491 32906590 PMC7559728

[B31] Oexle N. Sheehan L. (2020). Perceived social support and mental health after suicide loss. *Crisis* 41 65–69. 10.1027/0227-5910/a000594 31030548

[B32] Orgambídez A. Almeida H. (2020). Social support, role clarity and job satisfaction: a successful combination for nurses. *Int. Nurs. Rev.* 67 380–386. 10.1111/inr.12591 32436283

[B33] Oriol X. Miranda R. Bazán C. Benavente E. (2020). Distinct routes to understand the relationship between dispositional optimism and life satisfaction: self-control and grit, positive affect, gratitude, and meaning in life. *Front. Psychol.* 11:907. 10.3389/fpsyg.2020.00907 32528359 PMC7264816

[B34] Ostafin B. D. Proulx T. (2020). Meaning in life and resilience to stressors. *Anxiety Stress Coping.* 33 603–622. 10.1080/10615806.2020.1800655 32755239

[B35] Ren J. (2006). *Positive Psychology.* Shanghai: Shanghai Educational Publishing.

[B36] Russinova Z. (1999). Providers’ hope-inspiring competence as a factor optimizing psychiatric rehabilitation outcomes. *J. Rehabil.* 65 50–57.

[B37] Rustøen T. Cooper B. A. Miaskowski C. (2011). A longitudinal study of the effects of a hope intervention on levels of hope and psychological distress in a community-based sample of oncology patients. *Eur. J. Oncol. Nurs.* 15 351–357. 10.1016/j.ejon.2010.09.001 20870459

[B38] Sari S. V. (2019). Attaining career decision self-efficacy in life: roles of the meaning in life and the life satisfaction. *Curr. Psychol.* 38 1245–1252. 10.1007/s12144-017-9672-y

[B39] Scioli A. Ricci M. Nyugen T. Scioli E. R. (2011). Hope: its nature and measurement. *Psychol. Relig. Spiritual* 3 78–97. 10.1037/a0020903

[B40] Seguin C. (2019). A survey of nurse leaders to explore the relationship between grit and measures of success and well-being. *J. Nurs. Adm.* 49 125–131. 10.1097/nna.0000000000000725 30730404

[B41] Seligman M. E. Steen T. A. Park N. Peterson C. (2005). Positive psychology progress: empirical validation of interventions. *Am. Psychol.* 60 410–421. 10.1037/0003-066x.60.5.410 16045394

[B42] Sharkey C. M. Bakula D. M. Gamwell K. L. Mullins A. J. Chaney J. M. Mullins L. L. (2017). The role of grit in college student health care management skills and health-related quality of life. *J. Pediatr. Psychol.* 42 952–961. 10.1093/jpepsy/jsx073 28398538

[B43] Shiri S. Wexler I. Marmor A. Meiner Z. Azoulay D. (2020). Hospice care: hope and meaning in life mediate subjective well-being of staff. *Am. J. Hosp. Palliat. Care* 37, 785–790. 10.1177/1049909120905261 32052661

[B44] Snyder C. R. (2002). Hope theory: rainbows in the mind. *Psychol. Inq.* 13 249–275. 10.2307/1448867

[B45] Snyder C. R. Harris C. Anderson J. R. Holleran S. A. Irving L. M. Sigmon S. T. (1991). The will and the ways: development and validation of an individual-differences measure of hope. *J. Pers. Soc. Psychol.* 60 570–585. 10.1037//0022-3514.60.4.570 2037968

[B46] Steger M. F. Kashdan T. B. Sullivan B. A. Lorentz D. (2008). Understanding the search for meaning in life: personality, cognitive style, and the dynamic between seeking and experiencing meaning. *J. Pers.* 76 199–228. 10.1111/j.1467-6494.2007.00484.x 18331281

[B47] Steger M. F. Mann J. R. Michels P. Cooper T. C. (2009). Meaning in life, anxiety, depression, and general health among smoking cessation patients. *J. Psychosom. Res.* 67 353–358. 10.1016/j.jpsychores.2009.02.006 19773029

[B48] Sun F. K. Wu M. K. Yao Y. Chiang C. Y. Lu C. Y. (2021). Meaning in life as a mediator of the associations among depression, hopelessness and suicidal ideation: a path analysis. *J. Psychiatr. Ment. Health Nurs.* 10.1111/jpm.12739 Online ahead of print. 33559221

[B49] Taubman-Ben-Ari O. Weintroub A. (2008). Meaning in life and personal growth among pediatric physicians and nurses. *Death Stud.* 32 621–645. 10.1080/07481180802215627 18924291

[B50] Terry D. Peck B. (2020). Academic and clinical performance among nursing students: what’s grit go to do with it? *Nurse Educ. Today* 88:104371. 10.1016/j.nedt.2020.104371 32092601

[B51] Torregrosa-Ruiz M. Gutiérrez M. Alberola S. Tomás J. M. (2021). A successful aging model based on personal resources, self-care, and life satisfaction. *J. Psychol.* 155 606–623. 10.1080/00223980.2021.1935676 34165391

[B52] Tyer-Viola L. A. (2019). Grit: the essential trait of nurses during a disaster. *J. Perinat. Neonatal. Nurs.* 33 201–204. 10.1097/jpn.0000000000000416 31335843

[B53] Vainio M. M. Daukantaitë D. (2016). Grit and different aspects of well-being: direct and indirect relationships via sense of coherence and authenticity. *J. Happiness Stud.* 17 2119–2147. 10.1007/s10902-015-9688-7

[B54] Wang D. D. (2016). *Validation of the Short Grit Scale Among Chinese University and Secondary School Students.* Master’s thesis, China: Wuhan Sport University.

[B55] Wang X. Q. You Y. Y. Zhang D. J. (2016). Psychometric properties of meaning in life questionnaire Chinese version(MLQ-C) in Chinese university students and its relations with psychological quality. *J. Southwest University* 38 161–167.

[B56] Zeng J. Y. An F. R. Xiang Y. T. Qi Y. K. Ungvari G. S. Newhouse R. (2013). Frequency and risk factors of workplace violence on psychiatric nurses and its impact on their quality of life in China. *Psychiatry Res.* 210 510–514. 10.1016/j.psychres.2013.06.013 23850435

[B57] Zhang H. Zhao Q. Cao P. Ren G. (2017). Resilience and quality of life: exploring the mediator role of social support in patients with breast cancer. *Med. Sci. Monit.* 23 5969–5979. 10.12659/msm.907730 29248937 PMC5744469

[B58] Zhang Y. Cui C. Wang Y. Wang L. (2020). Effects of stigma, hope and social support on quality of life among Chinese patients diagnosed with oral cancer: a cross-sectional study. *Health Qual. Life Outcomes* 18:112. 10.1186/s12955-020-01353-9 32345317 PMC7189579

[B59] Zhao J. Zhang F. (2015). China is prepared to fight against emerging mental health disorders? *Int. J. Emerg. Ment. Health Hum. Resil.* 17, 628–634. 10.4172/1522-4821.1000244

[B60] Zimet G. D. Powell S. S. Farley G. K. Werkman S. Berkoff K. A. (1990). Psychometric characteristics of the multidimensional scale of perceived social support. *J. Pers. Assess.* 55 610–617. 10.1080/00223891.1990.9674095 2280326

